# Smelting of Scandium by Microwave Irradiation

**DOI:** 10.3390/ma10101138

**Published:** 2017-09-27

**Authors:** Satoshi Fujii, Shuntaro Tsubaki, Naomi Inazu, Eiichi Suzuki, Yuji Wada

**Affiliations:** 1 Department of Chemical Science and Engineering, School of Material and Chemical, Tokyo Institute of Technology, Tokyo 152-8550, Japan; tsubaki.s.aa@m.titech.ac.jp (S.T.); inazu.n.aa@m.titech.ac.jp (N.I.); suzuki.e.ac@m.titech.ac.jp (E.S.); yuji-w@apc.titech.ac.jp (Y.W.); 2 Department of Information and Communication System Engineering, National Institute of Technology, Okinawa College, Okinawa 905-2192, Japan

**Keywords:** scandium, smelting process, microwave irradiation

## Abstract

Scandium is being explored as an alloying element for aluminum alloys, which are gaining importance as high-performance lightweight structural alloys in the transportation industry. A few years ago, Sc was also found to be suitable for use in electrical devices. High-Sc-content ScAlN thin films have attracted significant attention because of their strong piezoelectricity. The piezoelectric response of ScAlN suggests that ScAlN thin films formed on a hard substrate would be suitable surface acoustic wave wideband filters for next-generation wireless communication systems. However, it is often difficult to use ScAlN thin films in MEMS devices—including acoustic ones—because of the extremely high price of metallic Sc, given the difficulty associated with smelting it. Here, we propose a novel process for smelting Sc metal by microwave irradiation. Sc metal was able to be obtained successfully from ScF_3_ through a microwave-irradiation-based carbon reduction reaction. The reaction temperature for this reduction process was approximately 880°C, which is half of that for the conventional smelting process involving reduction with Ca. Thus, the proposed microwave irradiation process has significant potential for use in the smelting of Sc metal.

## 1. Introduction

Scandium is classified along with yttrium and other lanthanides as a rare-earth (RE) element, has an atomic number of 21, an atomic weight of 44.96, and is the 31st most abundant element in the earth’s crust, exhibiting a Clarke number of 22 ppm [1]. Thus, scandium is more abundant than some well-known metals, such as tin and lead. Scandium is increasingly being used as an alloying element for aluminum alloys in the field of high-performance lightweight structural alloys [2,3]. Several advantages are realized when adding trace amounts of scandium to aluminum alloys. For instance, it has been reported that the alloy Al_20_Li_20_Mg_10_Sc_10_Ti_30_ is as light as aluminum, as strong as titanium, and as hard as a ceramic [4]. Because of these advantages, Al–Sc alloys are being produced commercially for use in aviation and aerospace components, bicycle frames, and baseball bats, among other applications. Sc is also used in metal halide lamps, known as mercury vapor lamps. Sc is added to these lamps, which are used in automobiles as a white light source [5]. A few years ago, it was also found that Sc can be used in electrical devices. High-Sc-content ScAlN thin films are attracting attention because of their strong piezoelectricity. Akiyama et al. found that the piezoelectricity of Sc_*r*_Al_1‒*r*_N thin films monotonically increases with the increase in the Sc concentration, *r*. The material’s piezoelectricity reaches its maximum at *r* = 43%, at which point the piezoelectric coefficient, *d*_33_, is five times that of pure AlN [6,7]. Hashimoto et al. reported that a surface acoustic wave (SAW) resonator based on the structure ScAlN/6H-SiC exhibited resonance *Q*, antiresonance *Q*, and *K*^2^ values of 340%, 240%, and 4.5%, respectively, at 3.8 GHz [8]. These values suggest that Sc*_r_*Al_1-*r*_N thin films formed on a hard substrate should be suitable as SAW wideband filters for next-generation wireless communication systems. However, it is often difficult to use ScAlN thin films in MEMS devices—including acoustic ones—because of the extremely high price of Sc metal, given the difficulty associated with smelting it.

Scandium is currently produced mainly through a thermal reduction method. Since scandium oxide as a raw material is extremely stable thermodynamically, scandium is produced by converting it into scandium fluoride, which is easily reducible, and reducing it at 1873 K using metallic calcium as the reducing agent [9]:

Sc_2_O_3_ + 6HF -> 2ScF_3_ + 3H_2_O, (1)

2ScF_3_ + 3Ca -> 3CaF_2_ + 2Sc. (2)

Since this process involves fluoridation, it is expensive and environmentally unfriendly. Furthermore, some of the calcium remains as an impurity. Several researchers have reported other processes for smelting Sc [10,11]. Harata et al. demonstrated that Al–Sc alloys can be produced directly via a calciothermic reduction reaction using CaCl_2_ as the flux and Al as the collector metal [11]. However, it is difficult to execute this process using a molten salt at high temperatures (approximately 1100 K). On the other hand, we were previously able to successfully obtain small amounts of magnesium metal by microwave irradiation. This process had a yield as high as 71% and consumed half of the energy used in the conventional process, which is known as the Pidgeon process [12]. In this report, we describe the application of the abovementioned process for the smelting of Sc metal by microwave irradiation and report the obtained results.

## 2. Experimental

The following two chemical reactions were utilized while using microwave radiation as the heat source:

Sc_2_O_3_ + CaH_2_ + Al -> Al-Sc alloy metal + CaO + H_2_, (3)

4ScF_3_ + 3C -> 4Sc + 3CF_4_. (4)

The starting material, Sc_2_O_3_, is converted into an Al–Sc alloy. For safety, we employed CaH_2_ instead of Ca metal.

## 3. Results and Discussion

**Figure 1:**
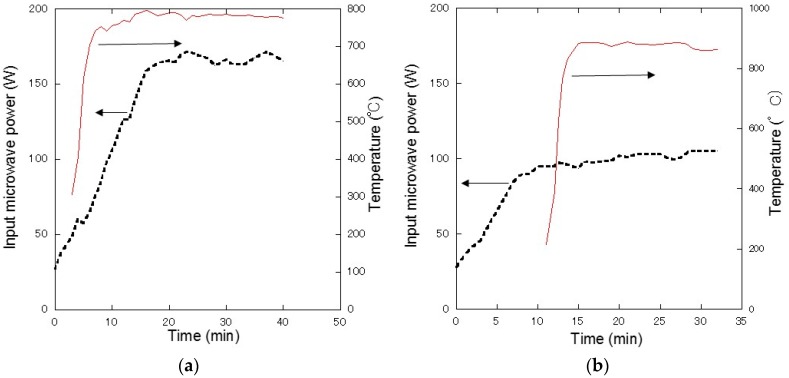
(**a**) Temperature of the crucible and microwave power as functions of time for the chemical reaction in Equation (3). The red lines represent the crucible temperature, and the black dotted lines represent the microwave power; (**b**) Temperature of the crucible and microwave power as functions of time for the chemical reaction in Equation (4). The red lines represent the crucible temperature, and the black dotted lines represent the microwave power.

Figure 1a shows the temperature of the crucible measured using an infrared sensor and the microwave power as functions of the time of the chemical reaction in Equation (3). During the process, the temperature exceeded 700 °C within 10 min, and the crucible was maintained at a temperature higher than 750 °C for 30 min. It was assumed that the sample temperature was more than 1000 °C when the crucible temperature was close to 800 °C. Figure 1b shows the temperature of the crucible measured using an infrared sensor and the microwave power as functions of the time for the chemical reaction in Equation (4). The temperature of the crucible reached 880 °C within 15 min, and was maintained at 880 °C for 15 min. In this case, in order to determine the sample temperature, a multiphysics simulation was also performed, and the sample temperature was determined to be the same as the crucible temperature.

**Figure 2:**
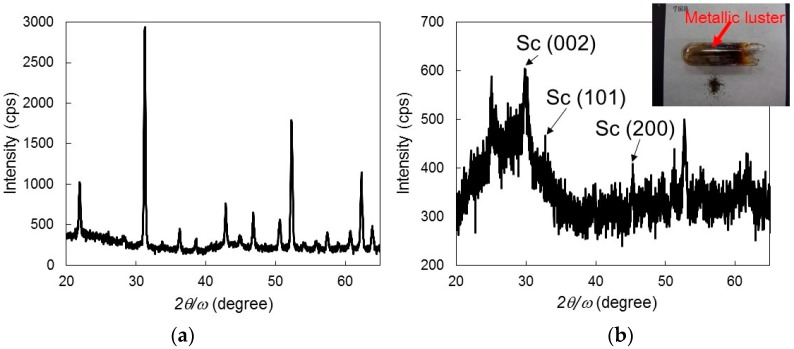
(**a**) XRD measurement results and photograph of the mixture of residues and products of the chemical reaction in Equation (3); (**b**) XRD measurement results and photograph of thin film formed on the glass tube after the chemical reaction in Equation (4).

It was difficult to separate the products from the residues after the chemical reaction in Equation (3). Figure 2a shows the results of X-ray diffraction (XRD) measurements (Rigaku, Tokyo, Japan) and a photograph of the mixture of the residues and products of this reaction. A few strong peaks related to calcium and a few weak peaks related to aluminum can be observed in the XRD pattern. However, it was difficult to determine whether there was a shift in the aluminum peak, which is indicative of the production of the Al–Sc alloy.

After the completion of the chemical reaction in Equation (4), a thin film with a metallic luster formed on the glass tube. Figure 2b shows the results of the XRD measurements and a photograph of the thin film formed on the glass tube. A few peaks can be observed in the pattern, even though their intensities are low. On the basis of JPCD references, the peak at 31° could be attributed to Sc (101). Thus, the thin film was confirmed to be Sc using XRD measurements. This is the first example of Sc metal obtained by smelting through microwave irradiation with carbon as a reduction material from ScF_3_. However, these results strongly suggest that the microwave irradiation process has immense potential for use in the smelting of scandium metal.

## 4. Discussion

**Figure 3:**
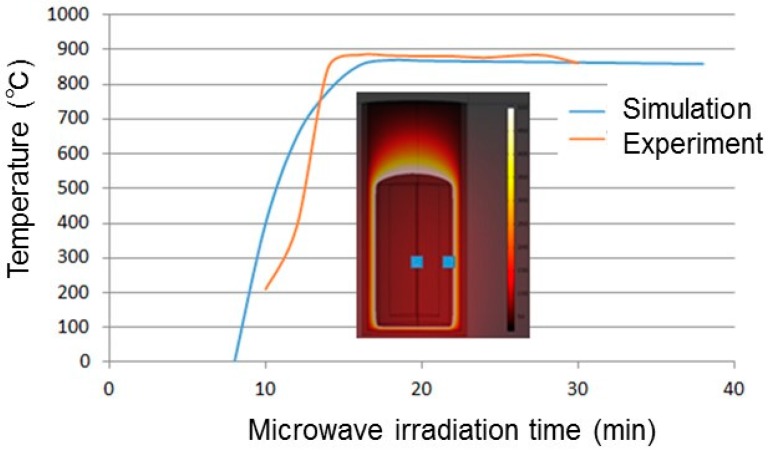
Observed temperature of the crucible wall (orange line) and calculated temperatures of the wall and inside of sample (blue lines, complete overlap) as functions of the microwave irradiation time.

Usually, the temperature of chemical reactions involving microwaves is lower than that in the case of conventional heating. However, in this case, the chemical reaction described by Equation (3) did not occur. The reason that the reaction in Equation (2) was able to proceed was that the internal energy of Ca is high, because Ca is in the gas phase, and the temperature of the reaction is high. On the other hand, the chemical reaction in Equation (4) occurs at 880 °C, which is approximately half of that for the conventional method. Figure 3 shows the results of a multiphysics simulation. The curves show the temperatures of the wall of the crucible and the inside of the sample as functions of microwave irradiation time. It can be seen that the two temperatures are the same, which was because the reactions were performed in a vacuum. Even though the reduction agents used in Equations (2) and (4) are different, the contact points of the particles probably undergo localized heating in the case of Equation (4). Haneishi et al. observed the specific local heating at the contact points during the microwave heating of silicon carbide spheres by in situ emission spectroscopy [13]. The same phenomena occurred in mixed particles of ScF_3_ and C. In this case, owing to the electric current at the contact points, the specific high temperature at the contact points was assumed to be generated up to the reaction temperature. However, the average temperature of the sample consisting of the mixed particles of ScF_3_ and C was low. Specific local nonequilibrium heating—so-called “hot spots”—has been suggested for this system.

## 5. Materials and Methods

**Figure 4:**
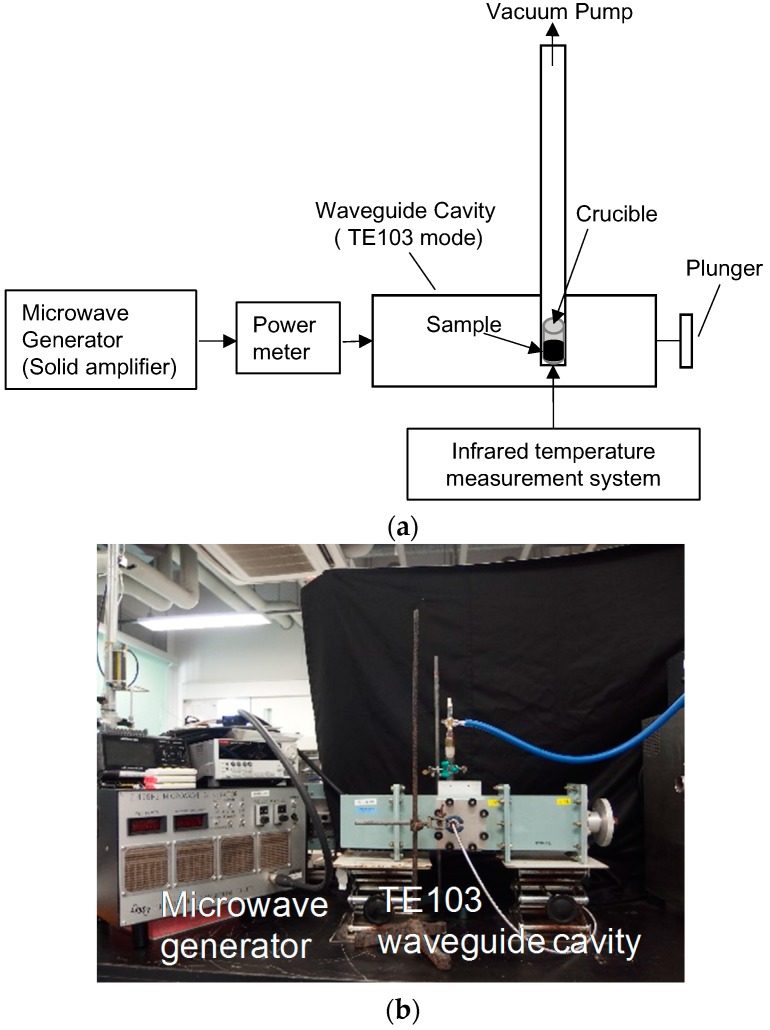
Experimental setup for metal smelting by microwave irradiation: (**a**) block diagram and (**b**) photograph of the system.

**Figure 5:**
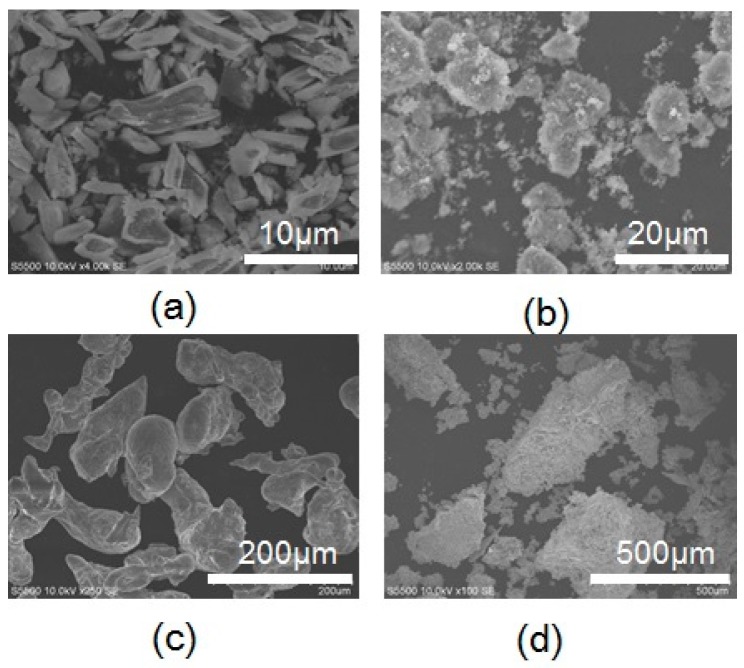
SEM images showing the particle morphologies of different powders: (**a**) Sc_2_O_3_; (**b**) ScF_3_; (**c**) Al and (**d**) CaH_2_.

Figure 4 shows the experimental setup consisting of a microwave generator with a phase-locked loop (PLL) oscillator, a power amplifier module (FSU-201VP-02, Fujidenpa Corp, Saitama, Japan), a plunger for impedance matching, a waveguide cavity with the TE103 mode, a rotary pump, a quartz glass tube, an infrared temperature measurement system (FTK9-R, Japan Sensor Corp, Tokyo, Japan), an alumina crucible (located in the TE103 cavity), and a sample (placed in the crucible). The input microwave power was the difference between the input power and reflected power, which were measured by the power meter incorporated in the amplifier module. Before the smelting process, the reflected power was adjusted to zero by the plunger in a short time. The temperature of the crucible was measured through a hole in the waveguide cavity by the infrared temperature measurement system. For the chemical reaction in Equation (3), a scandium oxide (Sc_2_O_3_) powder (average particle size: less than 10 μm), calcium hydride (average particle size: 100–500 μm), and an aluminum powder (average particle size: 100–300 μm) were mixed and packed in the alumina crucible to form a sample. The total weight of the mixture was 0.46 g, and the Sc_2_O_3_/CaH_2_/Al molar ratio was 0.15:0.96:0.27. The TE103 waveguide cavity has a standing electromagnetic wave in the cavity, and the maximum magnetic field in the cavity is obtained at two points. Usually, these maxima in the magnetic field are used to anneal conducting materials with microwave irradiation [14]. For the process described by Equation (3), a microwave power of 177 W was applied at the maximum magnetic field of the TE103 applicator, where the maximum field closer to the input port was employed, under low vacuum (10 Pa) and a crucible temperature of 780 °C. For the chemical reaction described by Equation (4), scandium fluoride powder (average particle size: less than 10 μm) and carbon particles (average particle size: 50 μm) were mixed and packed in the alumina crucible to form a sample. The total weight of the mixture was 0.68 g, and the ScF_3_/C molar ratio was 1:3. Figure 5 shows scanning electron microscopy (SEM) images of the various powders. For the process described by Equation (4), a microwave power of 100 W was applied at the maximum magnetic field of the TE103 applicator under low vacuum (10 Pa) and a crucible temperature of 880 °C. After the completion of the reactions, XRD measurements of the samples were performed to identify the Al–Sc metal alloy in the sample obtained by the reaction in Equation (3) and the Sc metal in the sample obtained by the reaction in Equation (4). Multiphysics simulations were performed using a finite element method called COMSOL in order to determine the reaction temperature at the center of the sample. Table 1 summarizes the parameters of each material used in the multiphysics simulation.

## 6. Conclusions

For the first time, we demonstrated that microwave irradiation can be used to smelt Sc metal. Using microwave radiation, we attempted to perform two chemical reactions. Instead of Ca, we used carbon as the reducing agent for fluoride reduction under microwave irradiation, the corresponding chemical reaction occurred at 880 °C, which is approximately half of the temperature for the conventional process. Generally, the price of carbon is cheaper than that of Ca, and carbon is also easier to use than calcium metal. Moreover, equipment with a heat capability below 1000 °C can be easily built in comparison with that with a heat capability over 1000 °C. Therefore, the reaction of carbon particles with scandium fluoride under microwave irradiation can potentially be used to smelt Sc metal.

## Figures and Tables

**Table 1:** Material parameters used in COMSOL.

Material	Conductivity (S/m)	Relative Permittivity	Relative Permeability	Thermal Conductivity (W/(m × K))	Density (kg/m^3^)	Heat Capacity (J/(kg × K))
ScF_3_ + C	1000	3.53–0.058j ^1^	1	118	2260	700
Glass (quartz)	0	4.2	1	10	2210	730
Alumina Crucible	0	1.8	1	5	3900	900

^1^ This value was determined via the perturbation cavity method. The other parameters are assumed from the COMSOL database.
